# The Effect of Propofol on Chronic Headaches in Patients Undergoing Endoscopy

**DOI:** 10.1155/2018/6018404

**Published:** 2018-01-21

**Authors:** David Giampetro, Victor Ruiz-Velasco, Ashlee Pruett, Matthew Wicklund, Robert Knipe

**Affiliations:** ^1^Penn State Hershey Medical Center, Department of Anesthesiology and Perioperative Medicine, Hershey, PA, USA; ^2^Department of Neurology, University of Colorado, Aurora, CO, USA

## Abstract

**Objective:**

This study determined acute and long-term effects of propofol administration in patients with severe headaches undergoing endoscopic procedures.

**Background:**

Approximately 13% of the US population is affected by migraines or severe headaches. The effect of propofol on headaches more than a few days after the intervention has not been explored.

**Methods:**

We employed a nonrandomized, prospective observational study that recruited patients with chronic headaches who received propofol from an outpatient endoscopy center for either upper or lower endoscopies. Patients completed the six-item Headache Impact Test (HIT-6) questionnaire prior to the procedure and 30 days after endoscopy. Additionally, the patients' response to propofol two days after endoscopy was assessed via phone.

**Results:**

The age of the participants (*n*=31) ranged from 20 to 70 years. The mean HIT-6 composite scores were significantly lower (*p* < 0.05) 30 days after propofol administration when compared to baseline scores. Upon stratification, 23 patients indicated an improved condition, 7 a worsened outcome, and 1 showed no change. Furthermore, mean scores were significantly lower (*p* < 0.05) in three HIT-6 questions pertaining to the severity of pain, daily activity, and frequency of lying down. Finally, the mean pain score obtained was significantly lower (*p* < 0.05) two days after procedure.

**Conclusions:**

The results of this suggest that propofol administration should be considered in treating chronic headaches. Double-blind studies are necessary to confirm these results.

## 1. Introduction

Migraines/severe headaches affect approximately 13% of the United States population [1–4]. Furthermore, women are three times more likely than men to have migraines/severe headaches [4]. Migraine headaches can be quite debilitating to the point where they have a detrimental impact on work productivity, physical functioning, lifestyle, psychological well-being, and leisure activities [3, 5, 6]. Migraine is a major cause of lost work days, costing American employers an estimated $13 billion per year [7].

Despite its ubiquity and association with substantial disability, migraine and other chronic headaches have historically been underrecognized and undertreated [8–10]. Chronic migraine, chronic tension-type headache, new daily persistent headache, and medication overuse headache account for the vast majority of chronic daily headaches [11].

Among studies conducted on the treatment of headaches, few have examined the effect of propofol (2, 6 di-isopropyl phenol) on the treatment of chronic headaches. The studies that have been performed exclusively examine the effect of propofol as an abortive agent for migraine headaches. Propofol is employed primarily as a rapid and short-acting intravenous medication for the induction of anesthesia and procedural sedation [12]. Pharmacologically, propofol is known to exert agonistic effects on gamma-aminobutyric acid (GABA) receptors. The neuropharmacology of propofol and the presumed multiple mechanisms of action upon various neurotransmitter systems in the brain, particularly GABA A receptor subtypes, may be responsible for the changes in the physiological condition of a migraine by activating these receptors, which results in significant pain reduction [1, 12]. Some studies have shown that propofol administration in patients prepared for epidural and other nerve blocks can have ameliorative effects. In some cases, headache severity was reduced almost completely [12]. In a study of 77 patients with intractable headache, Krusz et al. reported that the average reduction in headache intensity was 95.4%. Additionally, 63 of 77 patients reported complete abolition of their headache after an average of 20 to 30 minutes following intravenous treatment with propofol [12]. Another report analyzed the effectiveness of using low-dose propofol boluses for refractory chronic daily headaches. It was found that, in 17 of 18 subjects, propofol administration led to partial headache relief, though the patient group that dozed off or slept after propofol treatment reported the greatest relief [8]. It should be noted that the few studies employing propofol's effectiveness on headaches have focused primarily on migraine headaches as an abortive therapy with no study following patients beyond 3 days following propofol treatment. Therefore, the purpose of the present study was to determine the effect of propofol administration on patients with chronic headaches undergoing endoscopies in an outpatient center and if there is lasting effect beyond hours to a few days. Patient volunteers were asked to answer the Headache Impact Test (HIT-6) questionnaire, which was developed to assess how headaches influence the patients' daily activities and their capacity to function. Additionally, the pain scores for all volunteers were ascertained via phone two days after endoscopic procedure.

## 2. Methods

### 2.1. Subjects

Thirty-one participants between the ages of 20 and 70 with chronic headaches were recruited to participate in this nonrandomized prospective observational study. Of these thirty-one patients, twenty-three reported by history that they suffer from chronic migraine headaches, whereas the remaining eight only provided the response that they suffer from chronic headaches. It is unknown if a health care provider, such as a neurologist, diagnosed the individuals who indicated that they suffer from chronic migraines or if they simply refer to their chronic headaches as migraines without a formal diagnosis. We utilized a patient population undergoing screening and surveillance endoscopies, esophagogastroduodenoscopy (EGD) and colonoscopy, at an outpatient endoscopy center in Hershey, Pennsylvania, given the frequent use of propofol sedation in this population. Prior to endoscopy, patients received a phone call from the endoscopy center to obtain medical information and are asked a series of questions related to the endoscopy. In addition, every patient was asked if they suffer from chronic headaches. If they answered yes, they were asked if they would like to participate in a study exploring the relationship of anesthesia and the occurrence of headaches. Patients were then asked to fill out a baseline six-item Headache Impact Test (HIT-6) questionnaire.

The HIT-6 questionnaire is a validated measure of the impact of headache using 6 questions that include social-role functioning, pain, emotional distress and well-being, cognitive functioning, and vitality [5, 6, 13]. Additionally, the HIT-6 measures the severity of headache pain with an easily understood score that varies from 36 to 78, with scores greater than 60 indicating severe impact [13]. We administered the HIT-6 prior to endoscopy. At 30 days after endoscopy, we readministered the HIT-6 questionnaire via telephone interview. We also contacted patients by phone at two days after endoscopy with questions to assess response to propofol (Appendix 1). We asked participants, using a verbal numerical rating scale from 0 to 10, “What is your usual average headache pain score prior to your endoscopy?” and “What is your average headache pain score since your endoscopy?” We also asked, “How many headaches have you had since your endoscopy?” If we were unable to reach patients on call back dates (days 2 and 30 after endoscopy), we attempted to call patients back one day later and then excluded them from the study if we were unable to reach them.

Our inclusion criteria included subjects between the ages of 18 and 75 with chronic headaches. Subjects had to be capable of giving informed consent. Our exclusion criteria included subjects outside the age range of 18 to 75 and those not capable of providing consent.

### 2.2. Statistical Analysis

We employed the paired *t*-test to compare each question from the HIT-6 preendoscopy versus postendoscopy as well as the total HIT-6 score. Additionally, we compared subjects' usual average headache pain score to their headache pain score at two days after endoscopy using a paired *t*-test. GraphPad Prism was used for the statistical analysis of this study. All statistical tests were two-sided, and a *p* value less than 0.05 was considered statistically significant.

### 2.3. Study Ethics

The study was reviewed and approved by the Penn State Hershey Medical Center Institutional Review Board. Informed consent was obtained from all participants.

## 3. Results

In the current study, there were 32 participants, but one patient was excluded for failing to answer the questions on either day 30 or 31 postendoscopy per our study protocol. Of note, 29 of the subjects were female (Table 1). The mean age of the participants was 49.6 years, ranging from 20 to 70 years. Twenty-three patients underwent colonoscopy, 6 had an EGD, and 2 underwent both. All subjects received propofol ranging from 140 mg to 470 mg, with an average dose of 276 mg.

**Figure 1:**
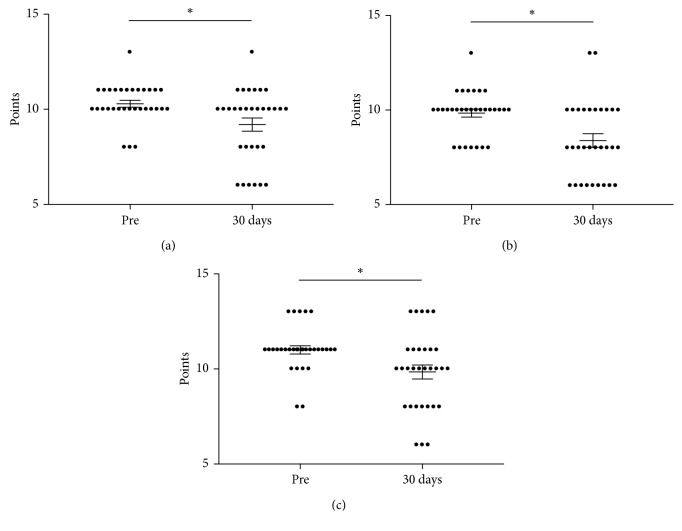
Summary dot plot with mean (±SEM) points of HIT-6 questions 1 (a), 2 (b), and 3 (c) obtained from 31 patients with chronic headaches undergoing endoscopy. The questionnaire was filled out before (pre) and 30 days after endoscopic procedure. ^∗^*p* < 0.05 employing a paired *t*-test.

Figures 1–1 show the summary dot plots for questions 1–3 of the HIT-6 questionnaire before and 30 days after endoscopy. Question 1, which concerns the severity of pain associated with headache, was significantly (*p* < 0.05, 95% CI: −1.85 to −0.34) lower 30 days after endoscopy. Furthermore, the mean number of points for question 2 was significantly (*p* < 0.05, 95% CI: −2.28 to −0.62) lower in patients 30 days after endoscopic procedure. The dot plot in Figure 1 depicts the mean points for question 3, which is indicative of the participants' likelihood to refrain from lying down. The plot indicates that the mean points were significantly (*p* < 0.05, 95% CI: −2.00 to −0.32) lower after receiving propofol.

**Figure 2:**
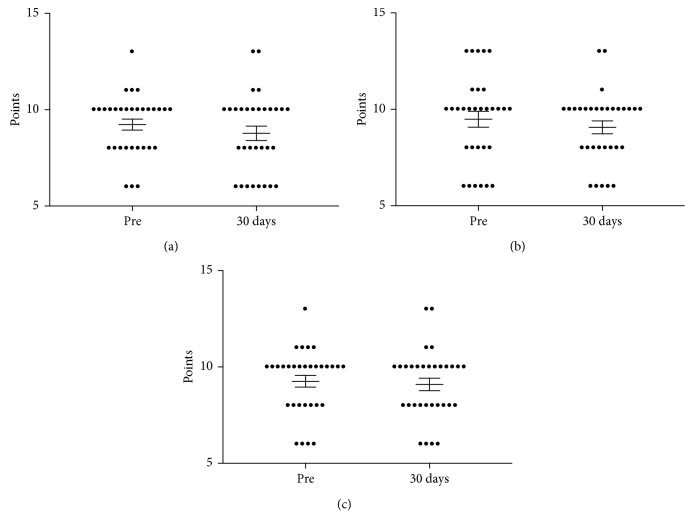
Summary dot plot with mean (±SEM) points of HIT-6 questions 4 (a), 5 (b), and 6 (c) obtained from 31 patients with chronic headaches undergoing endoscopy. The questionnaire was filled out before (pre) and 30 days after endoscopic procedure.

The dot plots shown in Figures 2–2 illustrate the mean scores for HIT-6 questions 4 through 6 before and 30 days after endoscopy. In these 3 questions, the scores were not significantly different. However, the frequency of answers with “rarely” or “never” increased following propofol administration.

**Figure 3:**
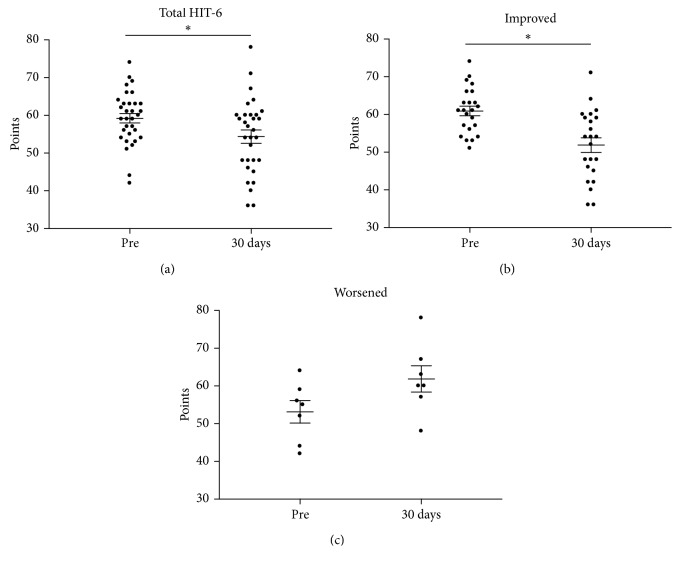
Summary dot plot with mean (±SEM) total points of all HIT-6 questions from the entire group of patients (*n*=31) with chronic headaches undergoing endoscopy (a). The plots in (b) and (c) depict the total points of patients sorted by decreased (*n*=23) and increased scores (*n*=7), respectively. A single patient with unchanged scores was not included in either category. ^∗^*p* < 0.05 employing a paired *t*-test.

Figure 3 shows the summary dot plot of the total HIT-6 questions before and 30 days after endoscopy. It can be observed that the scores were significantly (*p* < 0.05, 95% CI: −9.04 to −0.44) lower 30 days after propofol administration. The patients were also subdivided into headache outcome (i.e., improved, worsened, or no change). Figure 3 indicates that, in 23 patients with improved condition following propofol administration, the mean HIT-6 scores were also significantly lower. Of these 23 patients, 2 also received lidocaine 50 mg. On the other hand, 7 patients reported a worsening condition that was also significantly greater than that before propofol administration. One of these 7 patients also received fentanyl 25 mcg. One patient reported no change in pain condition. A comparison of the propofol dose administered between those with an improved condition (mean dose = 271 mg) and a worsened condition (mean dose = 294 mg) showed no significant (*p*=0.52) effects. When comparing the groups of improved HIT-6 score (*n*=23) versus worsened (*n*=7), there was no significant difference (*p*=0.52).

**Figure 4:**
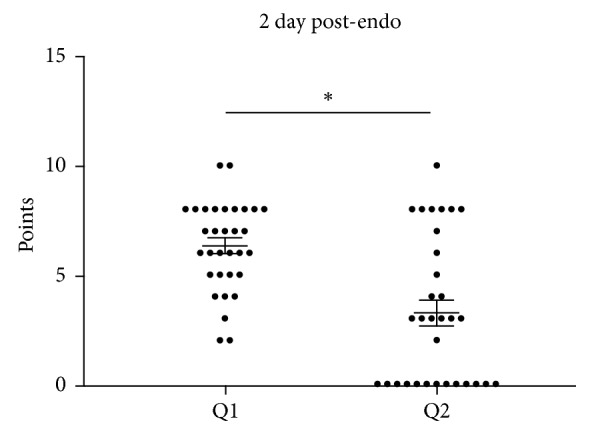
Summary dot plot with mean (±SEM) total points of two questions answered 2 days after endoscopic procedure from 31 patients with chronic headaches undergoing endoscopy. See text for details. ^∗^*p* < 0.05 employing a paired *t*-test.

We also determined headache pain scores 2 days after endoscopy of all participants that received propofol. The mean dot plot shown in Figure 4 indicates that there was a significant (*p* < 0.05, 95% CI: −4.34 to −1.78) decrease of the mean headache pain scores following propofol administration when compared to the baseline scores. It can also be observed that 13 subjects indicated a score of 0 following propofol administration.

## 4. Discussion

The present study, to our knowledge, is the first to assess the effect of propofol on chronic headaches in a patient population undergoing endoscopy. While most chronic headache studies employing propofol typically follow patients for three days or less, our study tracked patients' headache scores 30 days after endoscopic procedure. It should be mentioned that there is a single case report with one patient that performed a follow-up 72 hours after propofol administration [14]. All other studies examining the effect of propofol on headaches have exclusively focused on the abortive effect of propofol on migraine headaches, particularly ones refractory to conventional treatment, whereas this study sought to discern if propofol provides lasting benefit for chronic headache relief [8, 12, 14–22]. Our study is unique in that we did not limit our inclusion to chronic migraine, although 74% of participants indicated that they suffer from chronic migraines, but we do not know the accuracy of this diagnosis. Interestingly, despite allowing subjects to self-select for this study based on a single question, “Do you suffer from chronic headaches,” the average baseline HIT-6 composite score among our 31 subjects was 59.1 indicating that their degree of headache impact was substantial, with scores above 60 indicating very severe impact [13]. We found that subjects exposed to propofol at endoscopy reported an improvement of their headache 30 days after endoscopy compared to their baseline HIT-6 scores. This finding suggests that propofol plays a major role in lessening headache impact.

Krusz et al. previously reported the effectiveness of propofol as an abortive agent for migraine headaches in 77 patients. In that study, the mean propofol dose was 110 mg, and none of the participants received a dose sufficient to induce sleep. Other studies have employed propofol doses that ranged from 20 mg to 140 mg for abortive therapies [20]. On the other hand, in the present study, patients with chronic headaches received a mean propofol dose of 276 mg prior to undergoing endoscopic procedures. Further studies are necessary to elucidate whether there is a dose-dependent response to propofol that is different for maintenance therapy versus abortive therapy.

Soleimanpour et al. randomized 90 patients with migraine headache to receive propofol or dexamethasone for abortive therapy in the emergency department. The 45 patients receiving propofol were given 10 mg every 5 to 10 minutes up to a maximum of 80 mg. The mean reported pain was lower in the propofol group, and they achieved pain relief faster than the dexamethasone group. There are several studies, such as the aforementioned one, that clearly indicate the efficacy of propofol for abortive therapy. Until this study, little was known about the effect on patients' headaches beyond a few hours and in one patient, three days. Our study followed patients with a history of chronic headaches 30 days after propofol exposure, and we found that their headache impact was statistically lower at 30 days after propofol compared to baseline. Future work needs to be done to determine if there is an optimal dose for maintenance therapy of chronic headaches. It should be noted that two subjects received a dose of 50 mg of lidocaine in addition to propofol, which may have also contributed to the amelioration of their headache. Lidocaine has been shown to be effective for treating intractable migraines [23]. One such study showed that lidocaine at an average dose of 334 mg decreased the severity of refractory migraine headaches [24]. The two patients in our study who received lidocaine obtained one-sixth this amount.

Although there was no statistical difference for HIT-6 questions 4 through 6 between baseline scores for those questions and at 30 days after receiving propofol, there was a trend with more participants answering these questions with “rarely” or “never” at 30 days than at baseline (Figure 2). Another interesting observation is that all three of these questions start the same with, “In the past 4 weeks…,” which leads subjects to focus on a more specific timeframe rather than a global view of their headaches. This four-week period corresponds well with the time from receiving propofol to the follow-up HIT-6 questionnaire. Perhaps, these 30-day HIT-6 question scores are higher due to a possible “recency effect” where patients recall their experience closer to the end of the 30 days neglecting possible benefit early in this timeframe [25]. This could be suggestive of a wearing-off effect of the medication, provoking further inquiry of the possible need to redose propofol for chronic headaches sooner than 30 days.

This study has several limitations. This is a nonrandomized, observational study, which makes it vulnerable to biases including confounders associated with selection bias and lack of randomization. The difficulty with randomizing this population is that the overwhelming majority of patients receiving EGDs and colonoscopies are given propofol for sedation, which led to not having a control group. Another limitation may include the tendency for regression toward the mean. It is unknown whether these patients chose to participate due to worsened headache symptoms in the timeframe prior to study enrollment. Also, this study is limited by not subdividing the headache subtypes of participants. We allowed participants to self-define chronic headaches as a means of increasing enrollment, which is a limitation in and of itself. However, the average baseline HIT-6 composite score among subjects was 59.1, indicating that their degree of headache impact was substantial. Our limited contact with the subjects did not allow us to further elucidate their specific headache subtype. Future studies would benefit from performing a subset analysis of the headache subtypes to learn if propofol is more effective at treating certain subtypes than others.

Although the HIT-6 is insufficient to capture all aspects of headache, it measures the impact of headache in multiple domains of functioning, which in the context of those suffering from chronic pain, it is vital to understanding how patients are limited by their pain and also in gauging treatment success. The limitations of using the HIT-6 are that it fails to address other important aspects of headache such as triggers, headache days per month, pain quality, temporal factors, presence of aura, associated symptoms (e.g., photophobia, phonophobia, visual changes, scleral injection, etc.), and exacerbating and alleviating factors.

It should be noted that our study contained 2 male participants, indicative, perhaps, that women are more likely to participate in scientific studies than men [26]. Given that women are three times more likely than men to have migraines/severe headaches, the lower number of male subjects is not surprising and makes it difficult to discern whether gender differences affect outcome following propofol administration.

## 5. Conclusion

In summary, propofol infusions may be helpful for multiple headache types and as a maintenance medication, not just as an abortive agent for refractory migraine headaches. Our results demonstrated a statistically significant decrease in headache impact at 30 days following propofol administration for subjects with chronic headaches. Propofol infusion may have lasting beneficial effects in some patients with chronic headaches, which this study uniquely captures as all other studies on propofol use focused on acute abortive therapy. Additionally, subjects experienced significant improvement in their chronic headaches two days after receiving propofol when ascertained by a verbal numerical rating scale. A future direction for research includes stratification of different headache subtypes, such as migraine, tension headache, chronic daily headache, and trigeminal autonomic cephalgias, to see if there are differences in the effectiveness of propofol in treating particular headache subtypes. Additionally, further research in the form of double-blind studies needs to be conducted to ascertain if there is a dose-dependent response for abortive versus maintenance therapy.

## Figures and Tables

**Table 1:** Subject characteristics including the type of endoscopy and amount of sedation used. EGD = esophagogastroduodenoscopy.

Subject	Gender	Age	Type of endoscopy	Sedation and dose
1	Female	57	EGD	Propofol 150 mg
2	Female	55	Colonoscopy	Propofol 350 mg
3	Female	56	EGD	Propofol 150 mg
4	Female	57	Colonoscopy	Propofol 300 mg
5	Female	45	Colonoscopy	Propofol 250 mg
6	Female	55	Colonoscopy	Propofol 350 mg
7	Female	50	Colonoscopy	Propofol 180 mg
8	Female	54	EGD	Propofol 140 mg
9	Female	50	Colonoscopy	Propofol 240 mg
10	Female	53	Colonoscopy	Propofol 400 mg
11	Female	51	Colonoscopy	Propofol 250 mg
12	Female	55	Colonoscopy	Propofol 180 mg
13	Female	66	EGD/colonoscopy	Propofol 390 mg
14	Male	70	Colonoscopy	Propofol 300 mg
15	Female	58	Colonoscopy	Propofol 310 mg
16	Female	60	Colonoscopy	Propofol 250 mg
17	Female	20	Colonoscopy	Propofol 200 mg
18	Female	23	EGD	Propofol 300 mg, lidocaine 50 mg, glycopyrrolate 0.2 mg
19	Female	35	EGD/colonoscopy	Propofol 470 mg, fentanyl 25 mcg
20	Female	59	Colonoscopy	Propofol 280 mg, ephedrine 10 mg for low BP
21	Male	40	EGD	Propofol 200 mg
22	Female	29	Colonoscopy	Propofol 400 mg
23	Female	51	Colonoscopy	Propofol 320 mg
24	Female	57	Colonoscopy	Propofol 290 mg
25	Female	51	Colonoscopy	Propofol 270 mg
26	Female	44	Colonoscopy	Propofol 320 mg
27	Female	28	Colonoscopy	Propofol 230 mg
28	Female	50	Colonoscopy	Propofol 200 mg
29	Female	54	EGD	Propofol 300 mg, lidocaine 50 mg
30	Female	53	Colonoscopy	Propofol 270 mg
31	Female	52	Colonoscopy	Propofol 300 mg

## Abbreviations

GABA: Gamma-aminobutyric acid
EGD: Esophagogastroduodenoscopy
HIT-6: Headache Impact Test.

## Appendix

### Two-Day Postendoscopy Questions

1. What is your usual average headache pain score prior to your endoscopy on a scale of 0–10?
2. What is your average headache pain score since your endoscopy on a scale of 0–10?
3. How many headaches have you had since your endoscopy?
